# The Utstein Kloster and Its Role in Firearm Violence Policy

**DOI:** 10.5811/westjem.2021.2.52000

**Published:** 2021-05-04

**Authors:** Galen Adams

The Utstein *Kloster*^1^ (Norwegian for abbey) is Norway’s best-preserved medieval monastery. Utstein Abbey was consecrated in the late 13^th^ century and still functions today as a church and convent. The abbey (Figure 1) has also been the host site of several landmark analyses pertinent to emergency medicine, most notably on drowning,^2^ cardiac arrest,^3^ and trauma^4^ resuscitation. The Utstein style of analysis has been successfully developed as a multidisciplinary research framework for disaster medicine analysis.^5^ The Utstein style intentionally combines experts with a variety of scientific expertise in fields related to complex, multidimensional problems. Rather than a focus on narrow legal, policy, organizational, or sociological aspects of a disaster, the Utstein style borrows its multifactorial approach from Newtonian physics. The Utstein style analytical framework may be adapted to any multidimensional complex hazard such as firearm violence.

In Utstein style analysis, any potential disaster may be characterized as a *hazard* with stored potential energy. The *risk* of conversion of that potential energy to an *event* with kinetic energy occurs either at a statistically estimable rate (eg, hurricanes), or due to stochastic triggers (eg, terrorism). The r*isk* of an *event* becoming manifest can be modified through surveillance and prevention strategies, designed for each *hazard*. Should an *event* occur, the kinetic energy expended upon a population is termed i*mpact*. In the case of modern firearms, both the kinetic energy and the resulting *impact* are highly lethal.^6^ The v*ulnerability* of the population to the *impact* determines the *damage* to that population. After *impact* occurs, *damage* to the *vulnerable* population may be only be modified by timely active response and resources termed *resilience*. In the best case, the *prevention* of a *hazard* removes or disables its potential energy, rendering it harmless. If an *event* is allowed to *impact* a *vulnerable* population, the *damage* is mitigated by the *resilience* of the community. The Utstein style is an analytical heuristic, similar to the Haddon matrix,^7^ employed to separate and analyze the contribution of individual factors in the control of injury.

With respect to firearm violence, the citizen misuse of firearms would be the *hazard* in the Utstein framework. Along with Mexico and Guatemala, the United States (US) is one of three nations on earth that designates firearm possession as a Constitutional right and not a privilege. Therefore, the *hazard* of firearm violence cannot be *prevented* without amending the US Constitution. For this reason, our collective challenge is to find a better way to modify the *risk* of civilian misuse of firearms. State and local firearm ordinances represent an attempt at *risk* modification through a patchwork of restrictive and permissive strategies in which uniform enforcement is not possible. A wide variety of socioeconomic and cultural communities are overlaid on that patchwork of laws making the application of “gun control laws” confusing and contradictory. One law does not work in all places.

There are states, and in fact nations, that have high firearm ownership (ie, elevated *hazard)* and low rate of firearm violence (ie, low *event* occurrence), such as Hawaii, Idaho, Montana, Wyoming, or Switzerland. In contradiction, there are cities with both a high level of firearm regulation (ie, elevated *risk* mitigation*)* and a paradoxically high level of firearm violence (ie, elevated *event* rate) such as the District of Columbia or Chicago. The risk of firearm violence in the US resembles an archipelago of high-*risk* firearm violence islands with interspersed large zones of minimal *risk* oceans. One strategy does not fit all locations.

One possible explanation for these conflicting examples is perhaps that the problem is less about the firearm (ie, *hazard*) and more about the factors involved in motivating a citizen to misuse firearms. Because Second Amendment arguments lend themselves to primal emotions on both sides, too much energy is expended on the right of firearm possession vs dispossession (ie, *prevention*) and not enough on identifying and intervening in the factors leading up to the shooting or *risk* modification.

Americans accept the *risk* modification over *prevention* approach with motor vehicle accidents, swimming pool drownings, and air travel. Good policy and the avoidance of polarizing anger is guided by collecting data and using that data to analyze and modify *risk*. For example, the Haddon matrix has been used to modify the *risk* associated with motor vehicle travel. By separately analyzing pre-crash, crash, and post-crash factors, data-driven vehicle and highway designs are combined with regulatory, sociological, and psychological solutions to reduce motor vehicle injuries. Very few Americans are prohibited from driving a vehicle and the *risk* from motor vehicles crashes are mitigated by data-driven solutions.

With the acknowledgment that accidental firearm injury (eg, hunting accidents) is not included in this analysis, the issue of intentional firearm violence has at least four key categories:

- Suicide or self-harm
- Intimate partner, family, or business partner violence
- Criminal activity
- Mass shootings and assassinations

In each category, there are different factors that determine the *risk* of firearm violence becoming an *event.* Further, the target population has different *vulnerabilities*, with many different mitigation strategies. Like motor vehicle speed limits, one strategy does not fit all problem sets.

Suicide by firearm represents over one third of total firearm deaths in the US,^8^ and there are clear demographic groups (older White males), and predisposing circumstances (financial loss, family loss, loss of community stature) that correlate well with suicidality. These are stochastic triggers that indicate an individual’s likelihood of a firearm-assisted suicide and they are surveillable. A reporting system with data-driven intervention strategies such as peer outreach, psychological resources, or short-term firearm dispossession for identified high-risk individuals, may reduce the *risk* of a firearm-assisted suicide event in this category.

Intimate partner violence, family conflict, or revenge on business associates are significant subcategories for children and adults. Each of these subcategories involves some level of conflict or rejection, combined with a malign adjustment reaction. Similar to child abuse, or domestic abuse not involving firearms, there are higher risk individuals and precipitating events (eg, divorce, infidelity, family rejection, bankruptcy, larceny, etc) that are surveillable. Individuals undergoing these precipitating events may be screened and have data-driven resources provided such as personal, legal, and/or financial counselling. Higher *risk* individuals may be evaluated for short-term firearm dispossession and crisis counseling.

Given the cost of the judicial and prison systems in the US, criminal activity with firearm violence has perhaps the largest total resource allocation of the subsets. Great efforts have been made to predict criminal activity by better understanding the spatial, temporal, and perpetrator-victim associations of specific crimes. The risk modification of criminal behavior has received much less attention. If we assume that all people are born with more or less the same inclination to crime, then poor schools, gang activity, and systemic racial bias that produce disparate justice system outcomes are specific risk factors associated with poor and minority communities. These structural factors contribute to a loss of legitimate academic and/or economic opportunities and are a driver of criminal behavior. Consider, the US has 5% of the world’s population yet 25% of the world’s incarcerated population. Blacks and Hispanics represent 32% of the US population but 56% of the incarcerated population. While Blacks comprise 13% of the US population, 35% of those executed in the past 40 years are Black. Approximately half of those incarcerated will return to prison and 75% of formerly incarcerated people are unemployed.^9^ Simply stated, the imprisonment of poor and minority populations is not the answer to firearm violence. There is no doubt that the solution to systemic racial bias and its associated criminality is complex and will be difficult to overcome in the short term. That stated, to not address systemic racial bias will increase the *risk* of segments of our population to criminal behavior and associated firearm violence at a great cost in both lives and dollars.

While the category of mass shootings and assassinations is the most newsworthy and consistently evokes public outcry, it is actually 1–2% of the total firearm violence.^10^ Similar to criminal activity, great efforts have been made to mitigate mass shootings and assassinations, mainly through various dignitary protection strategies and the improvement of security for vulnerable sites (eg, schools, airports, public buildings). Like police funding for criminal activity, the mitigation of mass shootings and assassinations receives a large amount of the funding. Mass shooters and assassins do have distinct psychological profiles that occasionally include some elements of mental illness, being bullied, grievance, and perhaps the need for notoriety/revenge. Mass shooters are predominantly male and White and are often driven by a malign cause. Once again, these stochastic triggers are surveillable. Once identified, targeted resources directed to these vulnerable individuals with peer counseling, alternatives to violence, and firearm dispossession for recalcitrant individuals may decrease the incidence of these events.

For too long the US has avoided an injury control perspective, largely due to the Dickey Amendment of 1996,^11^ which prohibited the Centers for Disease Control and Prevention to collect these data. With the repeal of the Dickey Amendment in 2018, a new era of firearm injury control research is now possible. By adopting an injury control model such as the Utstein style analysis or the Haddon matrix, the factors associated with different categories of firearm violence may be identified and analyzed, and data-driven interventions developed and deployed. To remain in the status quo ensures that the US will remain a world leader in preventable firearm deaths. The door to a better way to control firearm injury has been opened. We have the ability to replace the overheated arguments on gun control with data-driven solutions for firearm violence.

A notional system to modify the issue of firearm violence is depicted in Figure 2. This Utstein style framework would require societal investment to identify and intervene in the risk factors of firearm violence. With data comes clarity and rational policies, tailored to each subset of problems and the locations and populations at *risk*. Informed with data, gun violence policy may improve, and firearm injuries may be reduced.

## Figures and Tables

**Figure 1:**
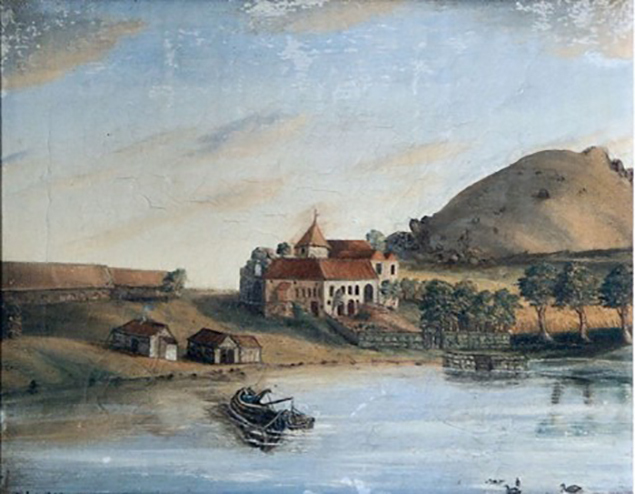
Anonymous 18th–19th century painting of Utstein Abbey (photographer Froda Inga Helland).

**Figure 2:**
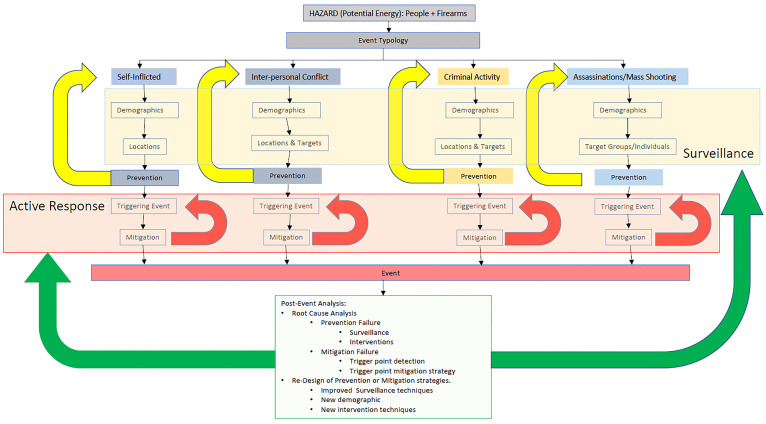
A notional Utstein framework to reduce firearm violence.
